# 68-year old woman with refractory cutaneous dermatomyositis

**DOI:** 10.31138/mjr.29.4.221

**Published:** 2018-12-18

**Authors:** Ioannis Antonopoulos, Stamatis-Nick Liossis

**Affiliations:** 1 Patras University Hospital, Department of Internal Medicine, Division of Rheumatology, Rion, Patras, Greece,; 2 Univerity of Patras Medical School, Rion, Patras, Greece

**Keywords:** Dermatomyositis, Vasculitis, Intravenous Immunoglobulin

## Abstract

Dermatomyositis is an idiopathic inflammatory disorder of the muscles associated with characteristic cutaneous findings. Herein we report a 68-year old woman who presented with dermatomyositis associated with painful vasculitic lesions on both hands, refractory to conventional treatment. Steroids, topical tacrolimus, antimalarials and intravenous cyclophosphamide were tried with no beneficial effect. Rituximab was also administered with no initial effect; soon afterwards, intravenous immunoglobulin was administered with good results. Some cases of cutaneous dermatomyositis may require trials of different therapies to identify the treatment regimen that produces satisfactory disease control.

## CASE PRESENTATION

A 68-year old woman with a previous history of hyper-thyroidism presented with fever, rash, hair loss, muscular weakness, dysphagia, dry cough and arthralgia. She denied weight loss or loss of appetite. She had undergone total hysterectomy 6 months ago for HPV-related cervical dysplasia without features of malignancy. She was a non-smoker and was treated with thiamazole for hyperthyroidism.

Clinical examination revealed diffuse facial erythema, a heliotrope eyelid rash and poikiloderma in a photosensitive distribution including the upper chest, in a “V-neck” configuration, the upper back (shawl sign) and the lateral thighs (holster sign). Gottron’s papules were noted over-lying both elbows, metacarpophalangeal, proximal and distal interphalangeal joints bilaterally. The palmar and lateral surfaces of the fingers were rough and cracked. She had painful ulcers of the buccal mucosa; especially the hard palate.

Painful vasculitic lesions were noted on the palmar aspects of her fingers on both hands, together with an ulcer. The lesions were so painful that the patient was unwilling to shake hands (*Figure 1A*). She had diffuse alopecia and arthritis of the wrists and metacarpophalangeal joints bilaterally. Neurological examination disclosed bilateral proximal upper and lower extremity weakness (muscle strength: 3/5) with preserved tendon reflexes and sensation. There were fine inspiratory crackles at both lung bases. There was no lymphadenopathy or organomegaly.

**Figure 1A.**
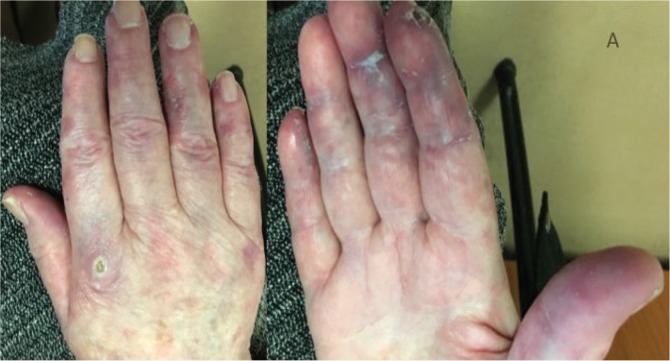
Gottron’s papules are noted overlying both metacarpophalangeal, proximal and distal interphalangeal joints bilaterally. Vasculitic lesions are also noted on the palmar aspects of the fingers on both hands together with an ulcer on the right index finger.

Laboratory work-up showed an elevated CRP (2.82mg/dL, normal < 0.8 mg/dL) and ESR (102 mm/h), leukopenia (WBC: 3850) and normochromic, normocytic anaemia (Hb=10.2 g/dL). Creatine kinase and thyroid-stimulating hormone were within normal limits. Testing for ANA was positive (1/640); anti-Ro was also positive and anti-Jo1 was negative. Anti-MDA-5, anti-NXP2 or anti-TIF1γ autoantibodies were not tested. Levels of C3 and C4 were normal. A diagnosis of dermatomyositis was made, and the patient was started on high dose steroids (predniso-lone 1mg/kg).

One month later, the patient presented at the rheumatology clinic without fever or arthritis, with improved muscle strength. Facial rash and poikiloderma were improved, but she still complained of troublesome vasculitic lesions of her hands. Chest and abdominal imaging, mammography and gastrointestinal endoscopy revealed no features of malignancy, and muscle biopsy was negative.

Skin biopsy disclosed myxoid degeneration of the dermis with mucus deposition and perivascular and intermediate lymphoplasmacytic infiltration together with mucus deposition around the vessels. The inflammatory infiltrate was located on the upper and lower dermis. Moreover, there was focal dropsical degeneration of the stratum basale and few melanocytes on the dermis. These features are not pathognomonic, but can be seen in dermatomyositis. Her pulmonary function testing revealed a restrictive pattern (FVC: 75%, FEV1: 88%, FVC/FEV1: 1.17). Based on the patient’s clinical improvement, steroids were tapered and hydroxychloroquine 200mg OD was added. Ten days later, the patient presented urgently with a rash exacerbation and was admitted for a single methylprednisolone i.v. pulse (1 gr). Hydroxychloroquine dose was doubled, and tacrolimus cream was added. One month later she presented with no rash improvement and another i.v. pulse of methylprednisolone was given with improvement. On subsequent follow-up visits, all other dermatomyositis symptoms kept improving, apart from the vasculitic rash. Steroid tapering resulted in flares. Two monthly intravenous pulses of cyclophosphamide were given, resulting in an initial clinical improvement followed by a subsequent flare. A cycle of rituximab was administered (1 gr, two weeks apart) with initial improvement and then a subsequent flare again.

Based on the refractory nature of cutaneous dermatomyositis, intravenous immunoglobulin (IVIG) was administered at a dose of 2gr/kg divided in 5 daily courses every month. A Few days before the third monthly IVIG cycle, our patient’s rash greatly improved (*Figure 1B*). Gottron’s papules and vasculitic lesions had nearly disappeared, as well as her poikiloderma lesions. Painful ulcers of the buccal mucosa healed, and new hair growth was evident in the scalp. Muscle strength was 5/5. She was given the third cycle of IVIG, steroids were tapered, tacrolimus cream was stopped, and hydroxychloroquine dose was reduced to 200mg once daily. She repeated a 2^nd^ cycle of rituximab at 6 months after the initial course. Today our patient is well on azathioprine (2mg/kg) and methylprednisolone 4mg once daily.

**Figure 1B.**
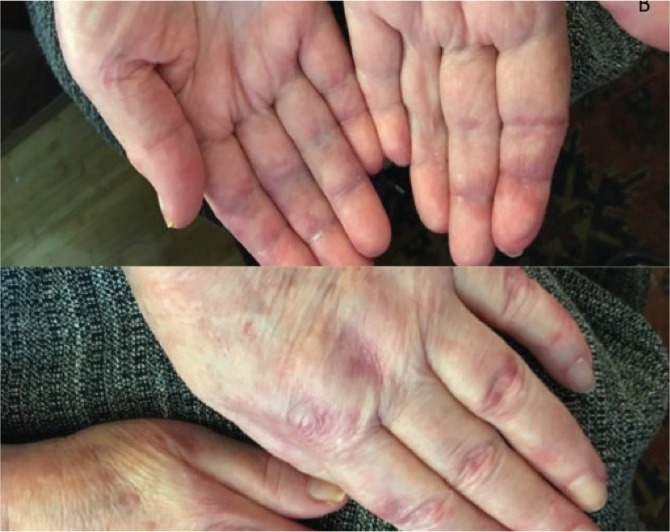
Improvement of Gottron’s papules and vasculitic lesions following 3 IVIG intravenous infusions.

## DISCUSSION

Dermatomyositis is an idiopathic inflammatory disease of the muscle, associated with cutaneous involvement. A careful work-up to exclude underlying malignancy is always mandatory. Patients who fail to respond to conventional therapies require more aggressive immunosuppressive or immunomodulatory treatment. Although a wide variety of medications have been used for the treatment of refractory cutaneous dermatomyositis, few formal studies have investigated the safety and efficacy of such agents. Patients who have failed conventional therapy (photoprotection, topical agents, hydroxychloroquine or methotrexate) are treated with mycophenolate mofetil (MMF)^1–4^ or intravenous immunoglobulin (IVIG);^5–7^ based upon the availability of small, uncontrolled studies. Intravenous immunoglobulin (IVIG) is an immune-modulating therapy with a wide variety of effects on the immune system. As a result, it is used successfully in different immune-mediated and systemic disorders, including the inflammatory myopathies. Dermatomyositis involves early accumulation of membranolytic attack complex (MAC) on the endomysial capillaries leading to capillary destruction, muscle ischemia and inflammation. IVIG inhibits this process by blocking the assembly and deposition of MAC on the endomysial capillaries through the formation of complexes between the infused immunoglobulin and C3b.^8^ Moreover, IVIG has been shown to downregulate the expression of the intercellular adhesion molecule (ICAM-I) on the endomysial capillaries, and the major histocompatibility complex class I (MHC-I) antigen on muscle fibres.^9^ The mRNA expression of TGF-beta1 has also been found increased in the muscles of patients with dermatomyositis. Following IVIG infusion, TGF-beta1 is downregulated and the TGF-beta1 mRNA decreases in the muscles of patients who successfully respond to treatment.^10^

However, the relatively favourable side effect profile, the oral route of administration, and the lower cost of MMF frequently lead clinicians to favour this drug slightly over IVIG. Moreover, rituximab has shown efficacy for refractory cutaneous DM.^11–12^

Our patient’s cutaneous vasculitis was refractory to steroids, topical tacrolimus, antimalarials and cyclophosphamide. Although the potential response to rituximab administration could be still considered as pending, treatment with IVIG was clearly effective within an acceptable 3-month period. In cases of dermatomyositis with refractory skin disease trials of different therapies (in combination or sequentially) are required to identify the treatment regimen that produces satisfactory disease control.
